# Supplementary material for: Inference of cell type-specific gene regulatory networks on cell lineages from single cell omic datasets
Source: Nat Commun. 2023 May 27;14:3064. doi: 10.1038/s41467-023-38637-9 (PMC10224950; doi:10.1038/s41467-023-38637-9)
Supplement: Supplementary file 3 — Reporting Summary [file 41467_2023_38637_MOESM3_ESM.pdf]

nature portfolio

Corresponding author(s): Sushmita Roy

Last updated by author(s): Apr 29, 2023

Reporting Summary

Nature Portfolio wishes to improve the reproducibility of the work that we publish. This form provides structure for consistency and transparency in reporting. For further information on Nature Portfolio policies, see our Editorial Policies and the Editorial Policy Checklist.

Statistics

For all statistical analyses, confirm that the following items are present in the figure legend, table legend, main text, or Methods section.

n/a	Confirmed	
☐	☒	The exact sample size (*n*) for each experimental group/condition, given as a discrete number and unit of measurement
☒	☐	A statement on whether measurements were taken from distinct samples or whether the same sample was measured repeatedly
☐	☒	The statistical test(s) used AND whether they are one- or two-sided<br>*Only common tests should be described solely by name; describe more complex techniques in the Methods section.*
☒	☐	A description of all covariates tested
☐	☒	A description of any assumptions or corrections, such as tests of normality and adjustment for multiple comparisons
☐	☒	A full description of the statistical parameters including central tendency (e.g. means) or other basic estimates (e.g. regression coefficient) AND variation (e.g. standard deviation) or associated estimates of uncertainty (e.g. confidence intervals)
☐	☒	For null hypothesis testing, the test statistic (e.g. *F*, *t*, *r*) with confidence intervals, effect sizes, degrees of freedom and *P* value noted<br>*Give P values as exact values whenever suitable.*
☒	☐	For Bayesian analysis, information on the choice of priors and Markov chain Monte Carlo settings
☒	☐	For hierarchical and complex designs, identification of the appropriate level for tests and full reporting of outcomes
☒	☐	Estimates of effect sizes (e.g. Cohen's *d*, Pearson's *r*), indicating how they were calculated

Our web collection on statistics for biologists contains articles on many of the points above.

Software and code

Policy information about availability of computer code

Data collection	No software was used to download publicly available datasets. The data were directly downloaded from GEO or databases.
Data analysis	The scMTNI code and custom scripts to process data and compute various validation metrics and perform dynamic network analysis are available at https://github.com/Roy-lab/scMTNI and Zenodo https://doi.org/10.5281/zenodo.7854535.<br>Custom scripts include shell scripts, python scripts, R scripts, and MATLAB scripts and we used R version 3.5.1, MATLAB version R2014b, and Python version 3.6.12 to perform data analysis. The scATAC-seq data was processed through CellRanger ATAC pipeline (Version 1.1.0). The simplified implementation of the pagoda pipeline for normalizing scRNA-seq data for depth and variance stabilization is available at https://github.com/Roy-lab/scMTNI/blob/master/Scripts/Integration/adjustVariance_depth_Generic.R. R package rliger version 1.0.0 was used to integrate scRNA-seq and scATAC-seq data, R script available at https://github.com/Roy-lab/scMTNI/tree/main/Integration/. To generate prior networks, we used MACS v2.1.0 to call ATAC-seq peaks to generate prior networks and used custom code for mapping TF binding peaks to genes, which is available at https://github.com/Roy-lab/scMTNI/blob/main/genPriorNetwork/. The custom scripts for evaluation based on AUPR and Fscore are available at https://github.com/Roy-lab/scMTNI/tree/master/Evaluation/. The custom scripts for dynamic network analysis are available at https://github.com/Roy-lab/scMTNI/tree/master/Network_Analysis/.

For manuscripts utilizing custom algorithms or software that are central to the research but not yet described in published literature, software must be made available to editors and reviewers. We strongly encourage code deposition in a community repository (e.g. GitHub). See the Nature Portfolio guidelines for submitting code & software for further information.

Data

Policy information about availability of data

All manuscripts must include a data availability statement. This statement should provide the following information, where applicable:

- Accession codes, unique identifiers, or web links for publicly available datasets
- A description of any restrictions on data availability
- For clinical datasets or third party data, please ensure that the statement adheres to our policy

The reprogramming scATAC-seq dataset generated in this study has been deposited to Gene Expression Omnibus (GEO) with accession ID GSE208620 (https://www.ncbi.nlm.nih.gov/geo/query/acc.cgi?acc=GSE208620).The scRNA-seq datasets for the same time points from Tran et al were downloaded from Gene Expression Omnibus (GEO) with accession ID GSE108222 (https://www.ncbi.nlm.nih.gov/geo/query/acc.cgi?acc=GSE108222).
The processed cluster-specific scRNA-seq matrices and the prior networks for reprogramming study are available at Zenodo https://zenodo.org/record/7879228.

The scRNA-seq data for human hematopoietic differentiation from Buenrostro et al were downloaded from Data S2 of Buenrostro et al. (https://ars.els-cdn.com/content/image/1-s2.0-S009286741830446X-mmc4.zip) and the scATAC-seq data were downloaded from Chen et al. (https://github.com/pinellolab/scATAC-benchmarking/tree/master/Real_Data/Buenrostro_2018). The scATAC-seq data are also available from GEO accession GSE96772 (https://www.ncbi.nlm.nih.gov/geo/query/acc.cgi?acc=GSE96772). The scRNA-seq data and the scATAC-seq data have been additionally uploaded to zenodo https://zenodo.org/record/7879228.The processed datasets for human hematopoietic differentiation are available at Zenodo https://zenodo.org/record/7879228.

The scRNA-seq (gene by cell) and scATAC-seq (peak by cell) data matrices for the human fetal hematopoietic differentiation data from Ranzoni et al were obtained from https://gitlab.com/cvejic-group/integrative-scrna-scatac-human-foetal. These are also available at ArrayExpress: E-MTAB-9067 (https://www.ebi.ac.uk/biostudies/arrayexpress/studies/E-MTAB-9067) for scRNA-seq and E-MTAB-9068 (https://www.ebi.ac.uk/biostudies/arrayexpress/studies/E-MTAB-9068) for scATAC-seq. The cluster-specific scRNA-seq matrices and the prior networks are available at Zenodo https://zenodo.org/record/7879228.

For mouse reprogramming study, the ChIP-based gold standard datasets were downloaded from ESCAPE (http://www.maayanlab.net/ESCAPE/) and ENCODE databases (https://www.encodeproject.org/). The Perturb-based gold standard networks were a union of the networks from LOGOF (loss or gain of function) based gold standard networks from ESCAPE database (http://www.maayanlab.net/ESCAPE/) and the networks from Nishiyama et al as the perturbation interactions.
The mouse gold standard datasets are available at Zenodo https://zenodo.org/record/7879228.

For human hematopoietic data, two gold standard datasets were a ChIP-based (Cus_ChIP) and a regulator knock down-based (Cus_KO) dataset in GM12878 lymphoblastoid cell line downloaded from Cusanovich et al. The third gold standard from ChIP-seq experiments in human hematopoietic cell types was downloaded from the UniBind database (https://unibind.uio.no/). The human gold standard datasets are available at Zenodo https://zenodo.org/record/7879228.

The source data underlying Figures 2-8, Supplementary Figures 2-3,5,7-10,12,14,15,17-20,22,24-28,20-29, 30-49, the cluster-specific scRNA-seq matrices and the prior networks for all datasets and scMTNI inferred consensus networks are available at Zenodo https://zenodo.org/record/7879228.

Human research participants

Policy information about studies involving human research participants and Sex and Gender in Research.

Reporting on sex and gender	N/A
Population characteristics	N/A
Recruitment	N/A
Ethics oversight	N/A

Note that full information on the approval of the study protocol must also be provided in the manuscript.

Field-specific reporting

Please select the one below that is the best fit for your research. If you are not sure, read the appropriate sections before making your selection.

☒ Life sciences ☐ Behavioural & social sciences ☐ Ecological, evolutionary & environmental sciences

For a reference copy of the document with all sections, see nature.com/documents/nr-reporting-summary-flat.pdf

Life sciences study design

All studies must disclose on these points even when the disclosure is negative.

Sample size	For scATAC-seq experimental data, the sample size is 6 biological samples, representing different time points of the reprogramming study. We chose 6 samples to analyze because these specific timepoints, along with MEFs and ESCs, provide enough comprehensive data on the various states and progression of cells during the reprogramming process. The size of each sample was randomly subsampled to the have the same

	number of input cells so that one particular sample didn't greatly outnumber the others and skew the results so that each sample had more equal representation in the analysis.<br><br>Network inference was done in a stability selection mode where we drew multiple sub-samples from the original data. The sample size is the number of cells in each subsample of the dataset for stability selection. Each subsample's size was set to 2/3 of the number of cells in the dataset. This number was determined to enable sufficient number of cells for each subsample.
Data exclusions	Cells with low read depth and genes with fewer than 5 or 20 measurements were filtered from downstream analysis. Some cell clusters were excluded if they had either no or too few scRNA-seq cells. Cluster C1 for the hematopoietic differentiation data from Buenrostro et al was removed from evaluation using the gold standards due to very few TFs overlapping the gold standards compared to the other cell clusters.
Replication	For scATAC-seq experimental data, we used one biological replicate for each sample to be analyzed. Previous experiments were conducted in which cells were reprogrammed using identical conditions and reagents (see Tran et al. 2019), and thus we set up the experiments in this paper with the assumption that one experimental replicate and one scATAC-seq submission for each sample reflects the same reprogramming time course observed in our previous experiments.<br><br>We have provided code, scripts, inputs and outputs from our experiments to enable replication of our study. All computational replications were successful using the same parameter settings, same computing environment and same input datasets.
Randomization	In the reprogramming scATAC-seq experiment, MEFs from a single embryo were randomly seeded at a density of 5,000 cells per well in 6-well plates.<br>For network inference in stability selection framework, data subsamples were generated by sampling uniformly at random from our full dataset.
Blinding	Blinding was not applicable to this study as no portion of this data could possibly be skewed based on participants' knowledge of the experiment. All cells from the reprogramming plates were collected during scATAC-seq submission, and the scATAC library prep and sequencing portions were performed by unbiased third parties who have no knowledge of any experimental details.

Reporting for specific materials, systems and methods

We require information from authors about some types of materials, experimental systems and methods used in many studies. Here, indicate whether each material, system or method listed is relevant to your study. If you are not sure if a list item applies to your research, read the appropriate section before selecting a response.

Materials & experimental systems

n/a	Involved in the study	
☒	☐	Antibodies
☐	☒	Eukaryotic cell lines
☒	☐	Palaeontology and archaeology
☐	☒	Animals and other organisms
☒	☐	Clinical data
☒	☐	Dual use research of concern

Methods

n/a	Involved in the study	
☒	☐	ChIP-seq
☒	☐	Flow cytometry
☒	☐	MRI-based neuroimaging

Eukaryotic cell lines

Policy information about cell lines and Sex and Gender in Research

Cell line source(s)	V6.5 ESCs (kind gift of Dr. Kathrin Plath)<br>Reprogrammable mouse embryonic fibroblast (MEFs) -primary isolation from pregnant mice at E13.5
Authentication	All ATCC lines are authenticated and pathogen free. V6.5 ESCs were validated in Sridharan et al., Cell, 2009. All reprogrammable MEFs were genotyped upon isolation.
Mycoplasma contamination	All new MEF isolations were tested immediately, and cells were routinely tested monthly for mycoplasma using e-Myco PLUS Mycoplasma PCR detection kit (Bulldog Bio, 25234).
Commonly misidentified lines<br>(See ICLAC register)	We do not use any of the commonly misidentified lines.

Animals and other research organisms

Policy information about studies involving animals; ARRIVE guidelines recommended for reporting animal research, and Sex and Gender in Research

Laboratory animals	The mice were housed in a facility that ran a 12hr light/12hr dark cycle, had an ambient temperature 72F, and maintained humidity between 20-50%. The mice used to generate the MEFs used for reprogramming were paired when they reached the appropriate breeding age of 6-8 weeks old. Male and female mice of breeding age from a mixed 129/Bl6 background that are homozygous for the

Oct4-2A-Klf4-2A-IRES-Sox2-2A-c-Myc (OKSM) transgene at the Col1a1 locus and heterozygous for the reverse tetracycline transactivator (rtTA) allele at the Rosa26 locus were time-mated, from which MEFs were isolated at E13.5. In this study, MEFs with a homozygous genotype for the OSKM transgene and rtTA allele were used for reprogramming experiments. Male neonatal human foreskin fibroblasts from ATCC (HFF-1 SCRC-1041) were used as feeders for our reprogramming cells. HFFs were passaged and expanded ~5 times prior to being irradiated.

Wild animals	No wild animals were used in this study
Reporting on sex	The process of somatic cell reprogramming is unaffected and is not influenced by the sex of the starting cell population, so the sex of the MEFs used in this experiment is unknown as it is irrelevant to the observed results.
Field-collected samples	No Field-collected samples were used in this study
Ethics oversight	Mice were maintained in agreement with our UW-Madison Institutional Animal Care and Use Committee (IACUC) approved protocol (ID M005180-R03).

Note that full information on the approval of the study protocol must also be provided in the manuscript.
